# Radial Basis Function-Sparse Partial Least Squares for Application to Brain Imaging Data

**DOI:** 10.1155/2013/591032

**Published:** 2013-05-13

**Authors:** Hisako Yoshida, Atsushi Kawaguchi, Kazuhiko Tsuruya

**Affiliations:** ^1^Department of Biostatistics, Graduate School of Medicine, Kurume University, Kurume 8300011, Japan; ^2^Department of Integrated Therapy for Chronic Kidney Disease, Graduate School of Medical Sciences, Kyushu University, Fukuoka 8118582, Japan; ^3^Biostatistics Center, Kurume University, Kurume 8300011, Japan

## Abstract

Magnetic resonance imaging (MRI) data is an invaluable tool in brain morphology research. Here, we propose a novel statistical method for investigating the relationship between clinical characteristics and brain morphology based on three-dimensional MRI data via radial basis function-sparse partial least squares (RBF-sPLS). Our data consisted of MRI image intensities for multimillion voxels in a 3D array along with 73 clinical variables. This dataset represents a suitable application of RBF-sPLS because of a potential correlation among voxels as well as among clinical characteristics. Additionally, this method can simultaneously select both effective brain regions and clinical characteristics based on sparse modeling. This is in contrast to existing methods, which consider prespecified brain regions because of the computational difficulties involved in processing high-dimensional data. RBF-sPLS employs dimensionality reduction in order to overcome this obstacle. We have applied RBF-sPLS to a real dataset composed of 102 chronic kidney disease patients, while a comparison study used a simulated dataset. RBF-sPLS identified two brain regions of interest from our patient data: the temporal lobe and the occipital lobe, which are associated with aging and anemia, respectively. Our simulation study suggested that such brain regions are extracted with excellent accuracy using our method.

## 1. Introduction

Recently, brain morphometry research has gained considerable attention for its proposed utility in the early detection of dementia and assessment of regional cerebral atrophy. Furthermore, several authors have reported an association between brain morphology and clinical characteristics such as age, chronic disease, and genetics [1–3] using magnetic resonance imaging (MRI) data. Voxel-based morphometry (VBM) is a commonly used technique for such analyses [4]. This method is based on general linear models with the values of each MRI voxel (in units of pixels, preprocessed for standardization) as a dependent variable and clinical characteristics (including the group indicator variable and covariates) as explanatory variables. However, this approach has some drawbacks which have been discussed by Davatzikos [5]. For example, a multiple-comparison correction requires several assumptions that are difficult to verify. An alternative to this approach is to use prespecified assemblies of voxels based on anatomical knowledge, which is known as a region of interest (ROI) approach. Therefore, the ROI approach requires the investigator to have precise and accurate knowledge of true anatomical boundaries. Moreover, variables need to be selected carefully in order to minimize the influence of irrelevant variables in the statistical model. We have taken a data-mining approach using an entire brain region and have used voxel intensity levels for the dependent variables and clinical characteristics (including patient background and blood test results) as explanatory variables.

There are two important statistical problems in the regression model that concern our use of large, complex data. The first is the selection of a set of relevant variables among a large number of both dependent and explanatory variables that are highly correlated. The partial least squares (PLS) regression, which was introduced by Wold [6], is a latent factor approach that is suitable for data with correlated variables. It has been used as an alternative approach to ordinary least squares (OLS) regression in ill-conditioned linear regression models that arise in several disciplines such as chemistry, economics, and medicine [7, 8]. Tibshirani has used PLS in neuroimaging [9]. The second problem is a problem in variable selection that often arises when the sample size *n* is much smaller than total number of variables (*p*; the so-called “large *p* small *n* problem”) for both dependent and explanatory variables. Utilizing the sparsity principle with *L*_1_-penalty has been promoted as an effective solution [9, 10]. This version of sparse PLS (sPLS) combines the *L*_1_-penalty and has been proposed by Lê Cao et al. [11] and Chun and Keleş [12]. The number of applications for this approach is steadily increasing in not only neuroimaging fields but also bioinformatics and chemometrics. This technique produces sparse, linear combinations of the explanatory variables and achieves both dimension reduction and variable selection simultaneously. The pioneering application of this method to brain imaging data has been used to investigate genetic polymorphisms and functional imaging data [3]. However, it is based on PLS regression in its symmetric (also called canonical) mode. In this paper, we consider the PLS in its regression mode based on the singular value decomposition (PLS-SVD). The difference lies in the fact that factors are orthogonal in the canonical mode, contrary to PLS-SVD, in which the loadings are orthogonal. The main concern in this approach is the restriction of analysis to prespecified brain regions. Using brain regions that have not been specified *a priori* would be more data-driven approach that may yield new and unexpected results, but such approaches typically introduce computational difficulties because of the large number of voxels to be analyzed. For this reason, we decided to combine this approach with a first step of dimension reduction on brain images using basis expansion.

In this paper, we propose a sparse PLS approach with basis expansion (RBF-sPLS; radial basis function-sparse partial least squares) and provide an application for real data using three-dimensional MRI brain scans with about a million voxels and 73 clinical characteristics from chronic kidney disease (CKD) patients. In addition, we conducted a simulation study to compare our proposed method with the original method. Our proposed, RBF-sPLS, prediction model with dimension reduction devices offers discriminant functions with excellent prediction performance in terms of sensitivity and specificity.

This paper is organized as follows.  Section 2 provided a discussion of three-dimensional MRI data and their preprocessing.  Section 3 states the proposed statistical methods. In Section 4, we report a simulation study for the characteristics of sPLS with basis expansion (RBF-sPLS) or without it (sPLS).

## 2. Data

### 2.1. Subjects

Between 2009 and 2012, we recruited 102 patients (mean age: 61 ± 11 years, 52% male, 48% female) with chronic kidney disease (CKD) to participate in our study. We examined brain volume using MRI scanning, and clinical parameters were measured on the same day. Patients were eligible if they were between 20 and 80 years old and had no prior history of brain injury such as stroke, traumatic brain injury, or brain tumor. The participant characteristics are shown in Table 1. Fifty-five percent of participants had a history of smoking (47 former and 9 current smokers). Blood pressure in the brachial artery was measured with the subjects in a sitting position after a 10 min rest. All patients provided informed consent. Kyushu University Institutional Review Board approved all procedures.

### 2.2. Image Data

Brain MRI was acquired from each subject using a 3.0 tesla MRI scanner of the same model. No major hardware upgrades occurred during the period. All subjects were scanned with identical pulse sequences: 124 contiguous, 3.0 mm thick axial planes of three-dimensional T1-weighted images (spoiled gradient recalled acquisition in steady state: echo time, 7 ms; flip angle, 30; voxel size, 1.02 × 1.02 × 1.5 mm).

We used the Statistical Parametric Mapping 8 software (SPM8, Wellcome Department of Cognitive Neurology, London, UK) to preprocess brain images. The segmentation algorithm from SPM8 was applied to every T1-weighted MRI scan to extract tissue maps corresponding to gray matter (GM), white matter (WM), and cerebrospinal fluid (CSF). The temporary common space of rigidly registered tissues is necessary as a starting point for the DARTEL algorithm. Next, the segmented tissues maps were used to create a custom template and associated warping fields were generated using the DARTEL template creation tool [4]. This tool estimates a best set of smooth, nonlinear deformations from each subject's tissues to their common average, applies the deformations to create a new average, and then reiterates until convergence.

## 3. Methods

### 3.1. Basis Expansion-Based Dimension Reduction

Suppose that we have *n* independent subjects {(**x**_*a*_, **s**_*a*_); *a* = 1,…, *n*}, where **x**_*a*_ ∈ ℝ^*p*^ are *p*-dimensional exploratory variable vectors (clinical characteristics) and **s**_*a*_ = (*s*_*a*_(**w**_1_),…,*s*_*a*_(**w**_*N*_))′ are *N*-dimensional vectors of brain images for the *α*th subject defined at points **w**_*i*_ ∈ *ℤ* ^3^  (*i* = 1,…, *N*). We used the radial B-spline function *ϕ*(·) [13] to reduce the dimension, which is represented as follows. For given *h* ≥ 0,

(1) ϕ(u)=14h2{h3+3h2(h−u) +3h(h−u)2−3(h−u)3,(u≤h),(2h−u)3,(h<u≤2h),0,(u>2h),

where *u* ≥ 0. We used the distance of these knots to define *h* as $h=\sqrt{3\times h_{0}^{2}}$, where *h*_0_ is the distance between adjacent knots. Then, the *N* × *q* dimension reduction matrix, **B**, is defined with the (*l*, *m*)-component being *b*_*lm*_ = *ϕ*(||**w**_*l*_ − **k**_*m*_||), where **k**_*m*_ ∈ *ℤ* ^3^  (*m* = 1,…, *q*) are equally spaced knots. Note that the value of *b*_*lm*_ is proportional to distance of **w**_*l*_ from **k**_*m*_. Therefore, **S** = (**s**_1_,**s**_2_,…**s**_*n*_)′ and the dependent variable matrix, **Y**, is constructed as

(2) $$\begin{array}{c} Y=SB. \end{array}$$

Thus, for PLS regression, our response and predictor matrices are **Y** = (**y**_1_,**y**_2_,…,**y**_*n*_)′ ∈ ℝ^*n*×*q*^ and **X** = (**x**_1_,**x**_2_,…,**x**_*n*_)′ ∈ ℝ^*n*×*p*^, respectively.

### 3.2. Sparse Partial Least Squares

Let **Y** denote an *n* × *q* dependent variable matrix and let **X** denote an *n* × *p* explanatory variable matrix. The core assumption of PLS regression is a latent decomposition of **Y** and **X** as follows:

(3) $$\begin{array}{c} Y=TQ^{\prime }+F,\;\;X=TP^{\prime }+E, \end{array}$$

where **T** is an *n* × *k* score matrix, *k* is the number of components, **P** and **Q** are *p* × *k* and *q* × *k* loading matrices, and **E** and **F** are *n* × *p* and *n* × *q* matrices of random errors.

The version of sparse PLS (sPLS) regression proposed by Lê Cao et al. [11] invokes singular value decomposition (SVD) of **M** = **X** ^**'**^ **Y** to yield the **M** = **U** **D** **V**′, where **U** is a *p* × *k* orthogonal matrix, **D** = diag⁡(*d*_1_, *d*_2_,…, *d*_*k*_) with *d*_1_ ≥ *d*_2_ ≥ ⋯ ≥ *d*_*k*_, and **X** is an **M** × **V** orthogonal matrix. Among variations of PLS regression, this is called PLS-SVD. From these, we can obtain the regression form **Y** = **X** **C** + **G** where **C** is a *p* × *q* regression coefficient matrix given by **C** = **U**(**P**′**U**)^−1^ **Q**′ and **G** is a residual matrix.

For ease of explanation for estimation, suppose that *k* = 1, then the objective function with a *L*_1_ penalization on **u** and **v**, which are column vectors of **U** and **V**, respectively, is given as follows:

(4) $$\begin{array}{c} L\left(u,v\right)=-u^{\prime }X^{\prime }Yv+\lambda_{X}{||u||}_{1}+\lambda_{Y}{||v||}_{1}, \end{array}$$

where *λ*_*X*_ and *λ*_*Y*_ are *L*_1_ penalization parameters for the weight vectors of matrices **X** and **Y**, respectively. This function is a minimized subject to ||**u**||_2_ = ||**v**||_2_ = 1. The amplitudes of *λ*_*X*_ and *λ*_*y*_ correspond to the increases and decreases of the number of **X** and **Y** variables, which contribute to the regression. For example, in the case of **X**, if the value of *λ*_*X*_ is large, then a large number of variables **X** would be selected. The same is the case for **Y**. Therefore, the sPLS concerns selection and modeling in a one-step procedure. This optimization problem is performed by the soft-thresholding function *g*_*λ*_(*y*) = sign⁡(*y*)(|*y*| − *λ*)_+_, where (*a*)_+_ = max⁡⁡(0, *a*) at each iteration of the NIPALS inner loop. Weight vectors **u** and **v** are computed using the following algorithm.

1. Initialize **u** and **v** using, for instance, the first pair of singular vectors of the matrix **X** ^**'**^ **Y** and normalize **u** ← **u**/||**u**||_2_ and **v** ← **v**/||**v**||_2_.
2. Until convergence of **u** and **v**:
  - for fixed **v**, $\hat{u}=g_{\lambda_{X}}(X^{'}Yv)$ and normalize $\hat{u}$ as in step 1;
  - for fixed **u**, $\hat{v}=g_{\lambda_{Y}}\left(Y^{'}Xu\right)$ and normalize $\hat{v}$ as in step 1;
  - $u=\hat{u}$, $v=\hat{v}$.
3. **t** = **X** **u**, **p** = **X**′**t**/**t**′**t**, and **q** = **Y**′**t**/**t**′**t**, where **t**, **p**, and **q** correspond to column vector of **T**, **P**, and **Q**, respectively.

For the general case of *k* > 1, the above algorithm is repeated for *k* times with the deflation step **X** ← **X** − **t** **p**′ and **Y** ← **Y** − **t** **q**′ as the fourth step. The final solution can be obtained as **T** = (**t**_1_,**t**_2_,…,**t**_*k*_), **Q** = (**q**_1_,**q**_2_,…,**q**_*k*_), and **P** = (**p**_1_,**p**_2_,…,**p**_*k*_), where the elements are obtained at each step among *k* steps.

### 3.3. Choice of Tuning Parameters

The choice of penalization parameters *λ*_*X*_, *λ*_*Y*_ and number of components *k* is important in model construction. We use a criteria called *Q* ^2^ proposed by Tenenhaus [14], which were used to select the number of components in the sPLS model in Lê Cao et al. [11] by performing cross-validation. We used 10-fold cross validation. Thus, our *Q* ^2^ has a functional form of *λ*_*X*_, *λ*_*Y*_, and *k* and is defined as

(5) $$\begin{array}{c} Q^{2}\left(\lambda_{X},\lambda_{Y},k\right)=1-\frac{\sum_{j\;=\;1}^{q}{\text{PRESS}}_{jk}}{\sum_{j\;=\;1}^{q}{\text{RSS}}_{j(k-1)}}, \end{array}$$

where PRESS_*jk*_ is the prediction error sum of squares and RSS_*jk*_ is the residual sum of squares for the *j*th-dependent variable and the PLS model with *k* components defined as follows. Let *κ*:  {1, 2, …, *n*} → {1, 2, …, 10} be an indexing function that indicates the partition to which observation *i* is allocated to *κ*(*i*)th part of the data by the randomization:

(6) $$\begin{array}{c} {\text{PRESS}}_{jk}=\sum_{i\;=\;1}^{n}{\left(y_{ij}-{\hat{y}}_{(-\kappa (i))j}\left(\lambda_{X},\lambda_{Y},k\right)\right)}^{2},\\ {\text{RSS}}_{jk}=\sum_{i\;=\;1}^{n}{\left(y_{ij}-{\hat{y}}_{ij}\left(\lambda_{X},\lambda_{Y},k\right)\right)}^{2}, \end{array}$$

${\hat{y}}_{{\left(-\kappa (-i)\right)}_{j}}\left(\lambda_{X},\lambda_{Y},k\right)$ is the predicted value for the *j*th-dependent variable from the sPLS model with penalization parameters *λ*_*X*_ and *λ*_*Y*_ and number of components *k* and estimated weight vectors from *κ*(*i*)th part of the data removed. That is, for any *i* subject, we predict that ${\hat{y}}_{(-\kappa (i))j}\left(\lambda_{X},\lambda_{Y},k\right)=x_{i}{\hat{b}}_{(-\kappa (i))j}\left(\lambda_{X},\lambda_{Y},k\right)$, where ${\hat{b}}_{(-\kappa (i))j}\left(\lambda_{X},\lambda_{Y},k\right)$ is the *j*th column of estimated regression coefficient matrix $\hat{B}$ from the sPLS model with penalization parameters *λ*_*X*_ and *λ*_*Y*_ and number of components *k* and  *κ*(*i*)th part of the data removed. ${\hat{y}}_{\kappa (i)j}\left(\lambda_{X},\lambda_{Y},k\right)$ is the predicted value with the same definition as ${\hat{y}}_{(-\kappa (i))j}\left(\lambda_{X},\lambda_{Y},k\right)$ except for the estimated weight vector from all available *n* subjects. We select the optimal set (*λ*_*X*_, *λ*_*Y*_, *k*) based on the maximization of *Q* ^2^(*λ*_*X*_, *λ*_*Y*_, *k*) among given candidates. This is implemented by the grid search.

## 4. Simulation Studies

In this section, we will illustrate the proposed methods in a simulation study. We demonstrate the impact of knot distance in affecting the representation of the results and clarify the advantage of dimension reduction by RBF by comparison to the method without basis expansion.

### 4.1. Data Sets

**Figure 1:**
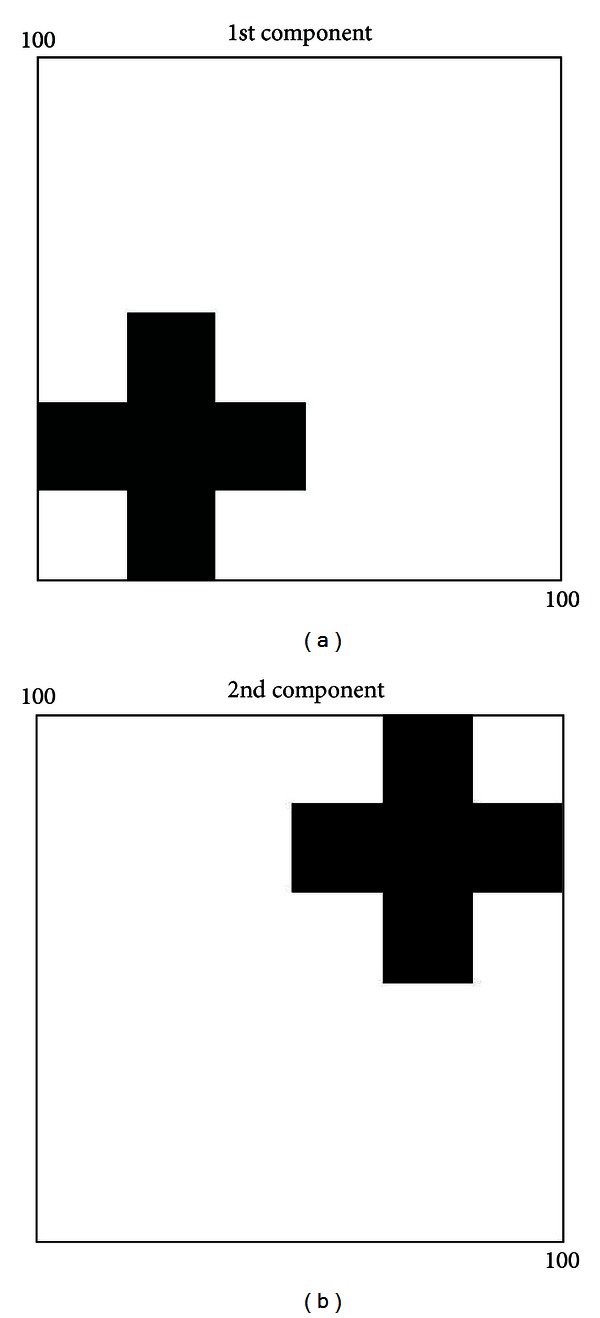
True grayscale images.

Consider *n* patients and *p* explanatory variables. We generated 100 datasets according to the following sPLS model with two components

(7) $$\begin{array}{c} X={\left(x_{1}^{},x_{2}^{},\ldots ,x_{n}^{}\right)}^{\prime },\;\;x_{j}∼\text{MVN}\left(0,\Sigma \right),\\ Y=TQ^{\prime }+F\;\text{with}\;T=XP^{-},\;\;F∼\text{MVN}\left(0,I\right), \end{array}$$

where MVN(0, Σ) denoted *p*-dimensional multidimensional normal distribution with zero mean and variance covariance matrix Σ. **P** = (**P**_1_,**P**_2_)  is the *p* × 2 matrix with **P**_1_ = **b**⨂(1_*p*/20_′,0_3*p*/20_′)′, **P**_2_ = **b**⨂(0_*p*/20_′,1_*p*/20_′,0_*p*/10_′)′, and **b** = (5,2,1,−2,−5)′, where ⨂ is the Kronecker product, 0_*c*_ is a *c*-dimensional vector with all elements 0, and 1_*c*_ is a *c*-dimensional vector with all elements 1. **Q** is the *q* × 2 matrix whose columns are vectorized true images displayed in Figure 1. The images can be thought of as 2D grayscale images with pixel intensities on the [0, 1] scale. The black pixels are set to 1 and the white ones are set to zero.

We performed this step in order to assess how much the performance of sPLS is influenced by the basis expansion and by the number of clinical parameters kept by the filter and to select the best pair of parameters. We provided a comparison with the original method (sPLS without the basis expansion) and also analyzed the impact of the distance between adjacent knots in our method for *h* = 2, 4, and  8. We tested our pattern of data set; *n* = 50/*p* = 40,  *n* = 50/*p* = 80,  *n* = 100/*p* = 40, and *n* = 100/*p* = 80 to replicate the sample size *n* of the CKD patients data set and the number of covariates *p*. The images **Y**'s were unfolded to obtain vectors of size *q* = 100 × 100 = 10,000.

### 4.2. Results

**Figure 2:**
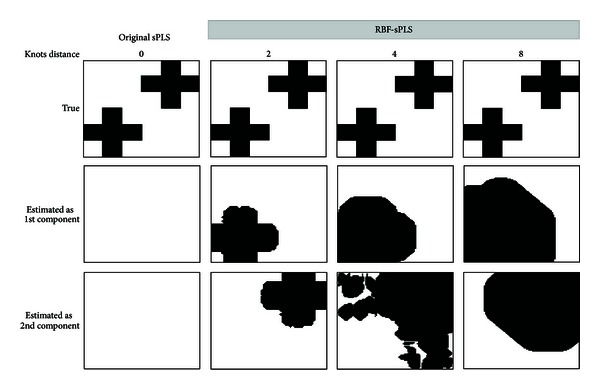
Binary images with threshold 0.95 for probability images from the simulation result of sPLS models with (knots distance = 2, 4, 8) or without basis expansion (knots distance = 0) for *n* = 50 and *p* = 40.

We estimated **P** and **Q** from simulated data by the method described in Section 3. All results yielded the correct number of components. We computed the probability images by averaging up the estimated **Q**'s from 100 datasets. The middle and bottom panels of Figure 2 display binary images converted from probability images with threshold 0.95 for the first and second components, respectively, in the case of *n* = 50/*p* = 40. The top of Figure 2 shows the combined true image. The result for sPLS without the basis expansion showed nothing at all because the maximum probability calculated was 0.7. On the other hand, the sPLS with the basis expansion with distance between knots *h* = 2 had a good shape, while for *h* = 4  and  8, the true image could not be reconstructed.

To assess how effectively the estimated model predicts each variable, sensitivity, specificity, and c-index = sensitivity − (1 − specificity) were computed and averaged over 100 sets. As shown in Table 2, the mean values of the c-index for the proposed method with *h* = 2 were relatively smaller than those for the method without the basis expansion and *h* = 4  and  8 in any cases of *n* and *p*. This indicated that the proposed method performed better than the original, and the distance between knots took on the smallest possible value.

## 5. Real Data Application

**Figure 3:**
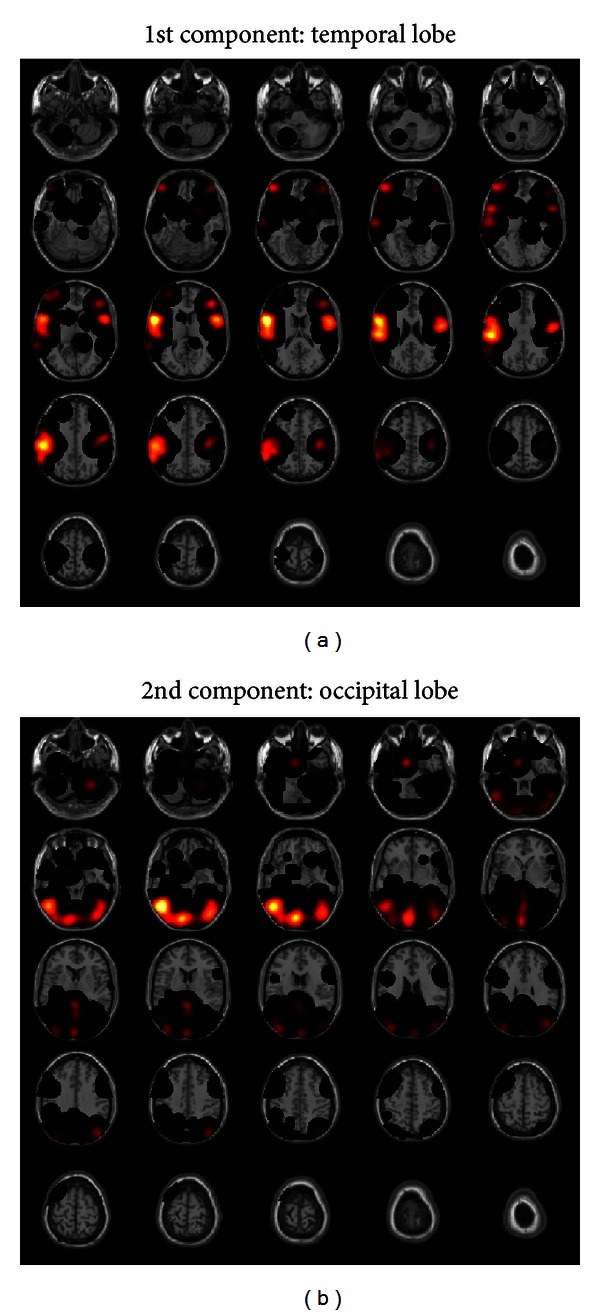
The brain region linked groups of each component.

We applied sPLS with basis expansion to our MRI dataset of the CKD patients described in Section 2. We assessed additional demographic and health-related variables, as well as laboratory data obtained on the same day. These data were used as covariates in our statistical analyses. The number of covariates is *p* = 73. Among the 2,122,945 (121 × 145 × 121) voxels for one subject, the voxels that represent brain regions are extracted, resulting in 839,089 voxels. The dimension of the basis function is *q* = 13,047 because of the 4-voxel ($h_{0}=4;\;\text{therefore},h=\sqrt{3\times 4^{2}}=6.93$) equal spacing knots. The number of components was selected as *k* = 2. The number of selected variables in the first component of **X** was 17, and 14 variables were in the second component. For **Y**, 785 and 947 variables were selected in the same manner.  Figure 3 shows the results by the axial view of brain. The left image shows the coefficient image estimated as the first component. Similarly, the right one shows the second component.

Our model revealed a relatively strong association between the bilateral temporal lobes and clinical markers of chronic kidney disease. The temporal lobes are one of the four main regions of the cerebral cortex. Structures of the limbic system, including the olfactory cortex, amygdala, and the hippocampus are located within the temporal lobes. The temporal lobes play an important role in organizing sensory input, auditory perception, language and speech production, and memory association and formation. These regions linked the 17 factors, in particular, age, sex, underlying disease (diabetes mellitus), smoking status, weight, serum albumin level, serum creatinine, total cholesterol, glucose, HDL-cholesterol, LDL-cholesterol, glycoalbumin, cholinesterase, number of red blood cell, whole parathyroid hormone, pulse wave velocity, and coronary artery calcification score.

The occipital lobes were selected by our analysis as the second component. The occipital lobes are positioned at the back region of the cerebral cortex and are the main centers for visual processing, involved in several functions of the body including visual perception and color recognition. This region linked the following factors: sex, body height, body weight, diastolic blood pressure, ratio of toe to brachial systolic blood pressure, total bilirubin, glucose, chloride, serum iron levels, number of red blood cells, hemoglobin, hematocrit, plasminogen activator inhibitor-1, and transferrin saturation.

The variables selected as the first component are considered to be the factors most closely related to aging and arterial stiffness, while those associated with the second region are more closely related to markers of anemia. The extent of atherosclerosis, calcification, and renal anemia are important complications in CKD patients. Recently, these factors have been suggested to be involved in brain atrophy and depressed cerebral oxygen metabolism [15, 16], but its mechanism remains to be elucidated. We also found a significant correlation between regional gray matter volume and hemoglobin level after adjusting for age, gender, residual renal function, underlying kidney disease, history of smoking, diastolic blood pressure, and LDL cholesterol level using multiple-linear regression methods [17]. In this analysis, we used only the whole gray matter volume as an objective variable, because multiple variables cannot be applied to conventional linear regression models, whereas the sPLS could select variables and modeling in a one-step procedure and use many objective variables.

## 6. Discussion

This paper describes that the radial basis function-sparse partial least squares (RBF-sPLS) technique was proposed and was applied to high dimensional brain imaging data. The original sPLS is a useful regression model to analyze data in which both dependent and explanatory variables are multivariate and correlated with one another. The most difficult problem in analyzing real brain data is the high dimensionality of these datasets. While prespecified regions were used in previous neuroimaging analyses, our method successfully handled a whole brain region following the basis expansion. The basis function has a spherical shape, but it was able to approximate the cross shape used in the simulation study. This would be expected because of the narrow spanned knots location. Thus, we set as close knots each other as possible in the real-data application, using 4-voxel equal spacing knots because computation using 2-voxel spacing was not possible. This method may be applicable to not only real brain data, but also general imaging datasets, because actual lesions would cause aggregates in adjacent voxels. Although the relative advantage of our proposed method was shown through the comparison between simulations run with and without the basis function conducted in the fair setting, further simulation studies with more realistic constraints are necessary. However, these simulations lie beyond the scope of the present paper and will be dealt with in the future. The significance of this study is to clarify the characteristics of RBF-sPLS presented visually for the analysis of imaging data.

We obtained clinically relevant findings about the relationship between aging, anemia, and brain morphology from the real-data application in our study. We are currently in the process of collecting longitudinal data and normal controls to expand this confirmatory evidence for future work. In summary, RBF-sPLS can help revealing the relationships between complex, large datasets, including brain imaging data.

## Figures and Tables

**Table 1:** The clinical characteristics about CKD patients' dataset.

	Mean ± SD
Number (male/female)	102 (49/53)
Age (years old)	61 ± 11
Diabetes (%)	27 (27)
BMI^a^ (kg/m^2^)	24.0 ± 3.9
SBP^b^ (mmHg)	124 ± 16
DBP^c^ (mmHg)	70 ± 12
eGFR^d^ (mL/min/1.73 m^2^)	39.8 ± 13.6
Smoker (*n *[%])	56 (56)

^a^Body mass index. ^b^Systolic blood pressure.

^c^Diastolic blood pressure. ^d^Estimate glomerular filtration rate.

**Table 2:** The result for sPLS without basis expansion and with, respectively, for 100 simulated data sets.

Knots distance	*P*	*n*	1st component			2nd component		
			Sensitivity	Specificity	C-index	Sensitivity	Specificity	C-index
Original sPLS: without basis expansion								
0	40	50	0.26	0.99	0.25	0.30	0.99	0.29
		100	0.34	0.99	0.33	0.39	0.99	0.38
	80	50	0.37	0.99	0.36	0.43	1.00	0.43
		100	0.39	0.99	0.38	0.44	1.00	0.44

RBF-sPLS: with basis expansion								
2	40	50	1.00	0.60	0.60	1.00	0.68	0.68
		100	1.00	0.86	0.86	1.00	0.87	0.87
	80	50	1.00	0.73	0.73	1.00	0.75	0.75
		100	1.00	0.84	0.84	1.00	0.88	0.88
4	40	50	1.00	0.29	0.29	1.00	0.13	0.13
		100	1.00	0.29	0.29	1.00	0.04	0.04
	80	50	1.00	0.27	0.27	1.00	0.13	0.13
		100	1.00	0.21	0.21	1.00	0.08	0.08
8	40	50	1.00	0.08	0.08	1.00	0.06	0.06
		100	1.00	0.05	0.05	1.00	0.00	0.00
	80	50	1.00	0.05	0.05	1.00	0.01	0.01
		100	1.00	0.02	0.02	1.00	0.00	0.00
